# Advancing Energy Efficiency in Smart Windows via Dual‐Responsive Electro‐Thermochromism

**DOI:** 10.1002/advs.77055

**Published:** 2026-08-07

**Authors:** Tongyu Chen, Bing Xu, Guolong Zhou, Wenting Wu, Bingkun Huang, Xiaohui Tang, William W. Yu, Pooi See Lee, Wu Zhang, Zhongyang Wang, Jingwei Chen

**Affiliations:** ^1^ School of Materials Science and Engineering Ocean University of China Qingdao China; ^2^ School of Materials Science and Engineering Nanyang Technological University Singapore Singapore; ^3^ School of Chemistry and Chemical Engineering Ministry of Education Key Laboratory of Special Functional Aggregated Materials Shandong Key Laboratory of Advanced Organosilicon Materials and Technologies Shandong University Jinan China; ^4^ Shandong Provincial Key Laboratory for Science of Material Creation and Energy Conversion Science Center for Material Creation and Energy Conversion Shandong University Qingdao China; ^5^ Institute of Medical Chips Ruijin Hospital Shanghai Jiao Tong University School of Medicine Shanghai China; ^6^ Ultrafast Optics and Nanophotonics Laboratory Department of Electrical and Computer Engineering University of Alberta Edmonton Alberta Canada; ^7^ State Key Laboratory of Metal Matrix Composites School of Materials Science and Engineering Shanghai Jiao Tong University Shanghai China; ^8^ Engineering Research Center of Marine Materials and Protection Technology Ministry of Education Key Laboratory of Marine Equipment Materials and Protection of Shandong Province Qingdao China

**Keywords:** electrochromism, energy saving, smart windows, thermochromism

## Abstract

Implementing dynamic smart windows is imperative toward the construction of net‐zero buildings. However, the prevailing ion (de‐)embedding‐based electrochromic (EC) or thermosensitive hydrogel‐based thermochromic (TC) windows fail to achieve large optical modulation across the solar spectrum owing to their optical absorption mechanisms. Herein, a dual‐responsive electro‐thermochromic smart window is designed and assembled by coupling an anodically coloring Prussian blue (PB) electrode with hydroxyl cellulose‐based hydrogel electrolyte (HPC). Through judicious formulation, the HPC hydrogel electrolyte affords large TC transmittance modulation (*∆*T ∼ 83% @633 nm), mechanical stability (strain = 514%), and a high ionic conductivity (σ = 62.58 mS/cm), enabling the PB electrode with a high coloration efficiency (CE = 108.21 cm^2^ C^−1^) and fast switching kinetics (t_b_ = 5 s, t_c_ = 6.8 s). As a result, the as‐assembled smart window features four distinct operating modes, exhibiting outstanding visible‐light T% modulation (ΔT_lum_ = 87.3%) and superior solar modulation capability (ΔT_sol_ = 50.5%), enabling a 35%–55% reduction in annual energy consumption in representative regions, and achieving annual energy savings up to 129 MJ m^−2^. This dual‐responsive smart window is an embodiment of coupled opto‐thermal regulation strategies toward building energy modulation, inspiring future efforts in energy‐efficient optoelectronics in the era of decarbonization.

## Graphical abstract

A durable and stable quasi‐solid‐state electro‐thermal dual‐responsive photothermal regulation device has been employed in advanced energy‐efficient smart windows. The devised device not only incorporates a thermochromic hydrogel characterized by high ionic conductivity, high optical transmittance, and a low LCST, but also comprises an electrochromic electrode with swift response and efficient performance. This device enables four distinct operational modes, with the superimposed mode demonstrating optimal energy efficiency, and integrates multifaceted functions including light modulation, thermal insulation, energy conservation, and energy storage.

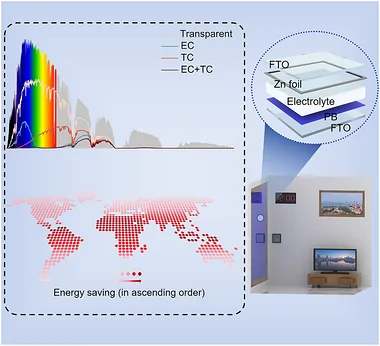

## 1 Introduction

Carbon neutrality has become a shared objective across the globe, driving efforts to simultaneously reduce carbon emissions and enhance energy efficiency. Nonetheless, continuous urbanization has led to increasingly prominent energy consumption. In developed countries, buildings account for approximately 30%–40% of total energy use [1, 2, 3, 4, 5, 6, 7], while heating, ventilation, and air conditioning are responsible for nearly 50% of building energy consumption [1, 3, 5, 8, 9, 10]. Moreover, energy use in cooling systems is projected to rise significantly over the next two decades. Windows, in particular, are considered the least energy‐efficient component of building envelopes due to their limited optothermal regulation capability, leading to increased indoor temperature and heavier reliance on air conditioning in summer, thereby substantially elevating overall energy consumption [11, 12, 13]. Therefore, dynamic windows capable of opto‐thermal regulation are promising avenues for advancing building energy efficiency [14].

Typical dynamic window technologies include electrochromic (EC) [2, 15], thermochromic (TC) [16], photochromic [17], and mechanochromic [18] platforms, among which EC and TC devices prevail due to their respective merits of active and adaptive opto‐thermal regulation. EC smart windows offer on‐demand regulation of light and heat upon application of a small electric bias, attracting broad interest in privacy windows, aircraft portholes, automotive sunroofs, and switchable sunglasses [19, 20, 21, 22], while TC windows respond automatically to temperature changes without external energy input, offer a sustainable approach for adaptive solar regulation [10, 23, 24].

Electrochromic devices (ECDs) are dominated by ion (de‐)embedding and reversible metal electrodeposition (RME)‐based mechanisms. RME‐based ECDs can achieve extremely low transmittance (T%) across the solar spectrum when a thin metallic layer is plated [1, 2, 7], but are still challenged by reversibility, scalability, and stability. Comparatively, ion (de‐)embedding‐based ECDs achieve fast response and excellent stability [25, 26, 27]. As for TC smart windows, vanadium dioxide (VO_2_) and thermosensitive polymer hydrogels are typical candidates. VO_2_ undergoes a monoclinic to tetragonal phase transition at elevated temperature with increased near‐infrared (NIR) absorbance [3, 28], but suffers from relatively high intrinsic transition temperature and low visible (VIS) light T%. Instead, thermosensitive polymer hydrogels, e.g. poly(N‐isopropylacrylamide) (PNIPAm) [29, 30] and hydroxypropyl cellulose (HPC) [31, 32] possess appropriate lower critical solution temperature (LCST) and higher VIS light T%. Notably, compared to PNIPAm with toxicity issues [33] and poor stability [34, 35, 36], environmentally benign cellulose are favored. At temperature below LCST, the hydrophilic hydroxyl groups on HPC molecular chains form hydrogen bonds with water, rendering a swollen state with high T%. At temperature higher than LCST, the increased hydrophobicity of HPC molecular chains leads to phase separation and aggregation with increased light scattering, rendering an opaque state with low T% [31, 32].

The active EC and adaptive TC devices are leading candidates for smart windows. However, ion (de‐)embedding‐based ECDs struggle to maintain consistently low T% across the VIS wavelength due to their intrinsically spectrally selective absorption mechanisms [37, 38], whereby HPC‐based TC devices can hardly block solar radiation in the NIR range due to the scattering‐based optical modulation mechanism [33, 39]. Therefore, integrating dual‐responsive EC‐TC platform with large optical modulation is a promising solution toward energy‐efficient smart windows [40, 41, 42].

In this regard, by coupling anodically coloring EC Prussian blue (PB) electrode with a temperature‐responsive HPC‐based hydrogel electrolyte, a dual‐functional EC‐TC smart window (termed as PH window) was assembled, exhibiting four distinct operation modes that overcome the limitations of standalone EC or TC technologies. With well‐tuned HPC and acrylamide (AM) ratios, the optimized hydrogel electrolyte simultaneously achieves a larger TC transmittance modulation (*∆*T) of 83.5% at 633 nm, a low LCST of 30°C, excellent mechanical stability (strain = 514%), and high ionic conductivity (σ = 62.58 mS/cm) at room temperature. As a result, the as‐assembled PH window features a VIS transmittance modulation (ΔT_lum_) of 87.3% and a solar modulation (ΔT_sol_) of 50.5%, reduce energy consumption in urban regions by up to 35%–55%, and achieves energy savings up to 129 MJ m^−2^. The outstanding optical modulation capability and high energy efficiency presented in this PH window provide a viable pathway and offer valuable insights in advancing building energy efficiency via coupled opto‐thermal regulation technologies.

## 2 Results and Discussion

## 3 Demonstration of the PH Smart Window

Dual‐responsive PH windows are favorable for building energy savings. Figure 1a illustrates the working mechanism and configuration of the assembled PH smart window, which features a sandwich structure consisting of two fluorine‐doped Tin Oxide (FTO)‐coated glass substrates enclosing an EC PB electrode, a TC HPC hydrogel electrolyte, and a zinc foil counter/reference electrode. The dual functionality of the PH smart window stems from the integration of active, on‐demand EC control and passive, temperature‐adaptive thermochromism. Figure 1b depicts the four operation modes, namely a transparent state, a blue colored EC state upon voltage application, an opaque milky TC state upon heating, and a combined milky blue EC‐TC state under simultaneous heating and voltage application. As depicted in Figure 1c, PH smart windows in either EC or TC state show notably high T% in the VIS or the NIR wavelengths. Nonetheless, PH smart windows in the EC+TC state can effectively block most of the VIS and NIR wavelengths, thereby showing larger T_lum_ and T_sol_ modulation than standalone EC or TC windows (Figure 1d), rendering higher opto‐thermal regulation capabilities favorable for advancing building energy efficiency.

**FIGURE 1:**
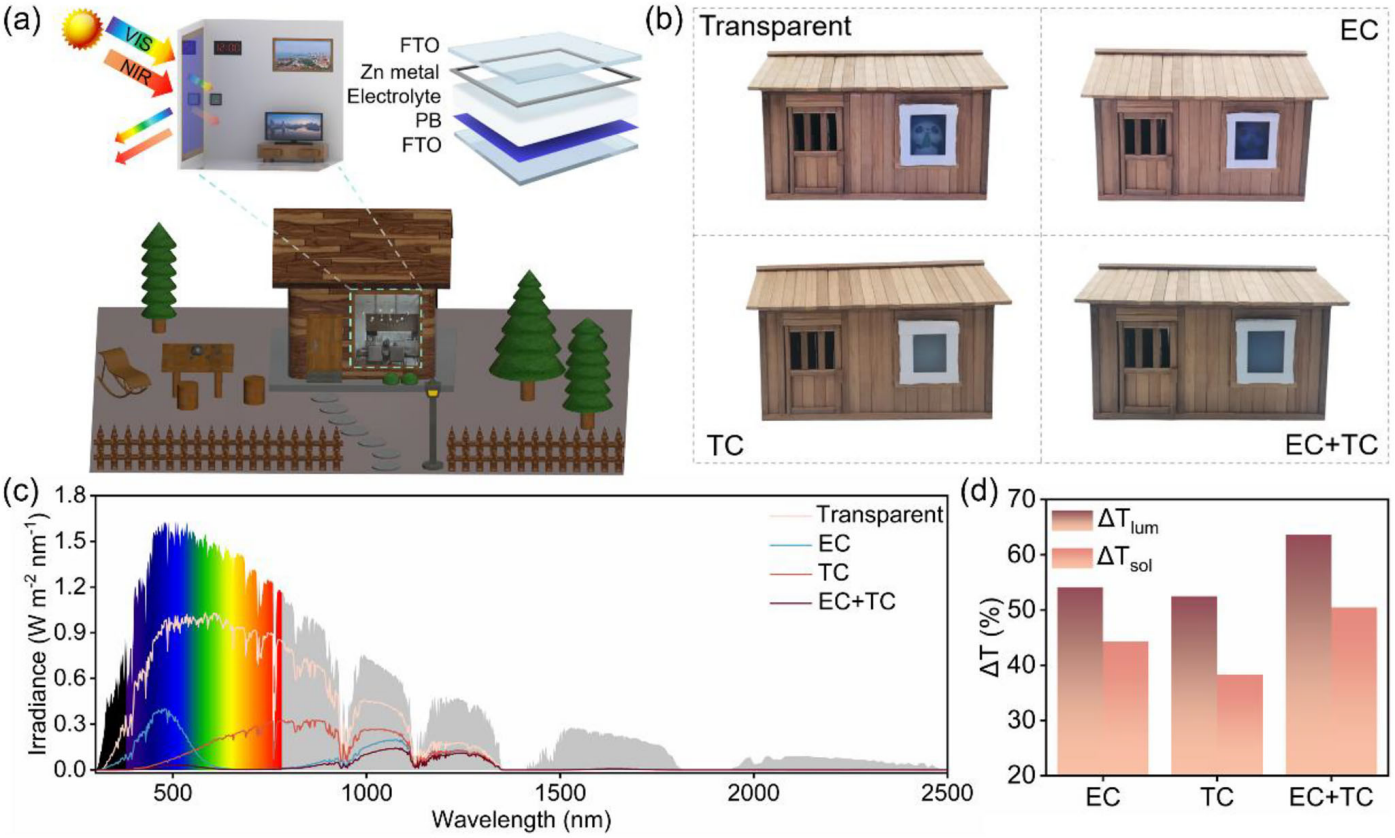
Mechanism, operation modes and performance of dual‐response electro‐thermochromic smart window. (a) Schematic illustration of the configuration, (b) practical demonstration of the four operating modes and (c) corresponding solar irradiation blocking performance of the PH smart window. (d) Comparison of T_lum_ and T_sol_ modulation of the PH window at different states.

## 4 Formulation of Thermochromic HPC Hydrogel Electrolyte

The thermo‐responsive behavior of HPC was ascribed to the coexistence of hydrophilic hydroxy groups and hydrophobic methyl groups. As illustrated in Figure 2a, below the LCST, the hydrophilic hydroxy groups engage extensively in hydrogen bonding with water molecules, promoting a well‐solvated, extended chain conformation that ensures high T%. When heated above the LCST, the attenuation of hydrogen bonds and the enhanced hydrophobic interactions among methyl side chains trigger a collapse of the extended structure, rendering an opaque milky state [31]. This reversible transparent‐to‐opaque transition lays the foundation for the HPC hydrogels in smart TC window applications, enabling adaptive dynamic optothermal modulation (Figure 2b).

**FIGURE 2:**
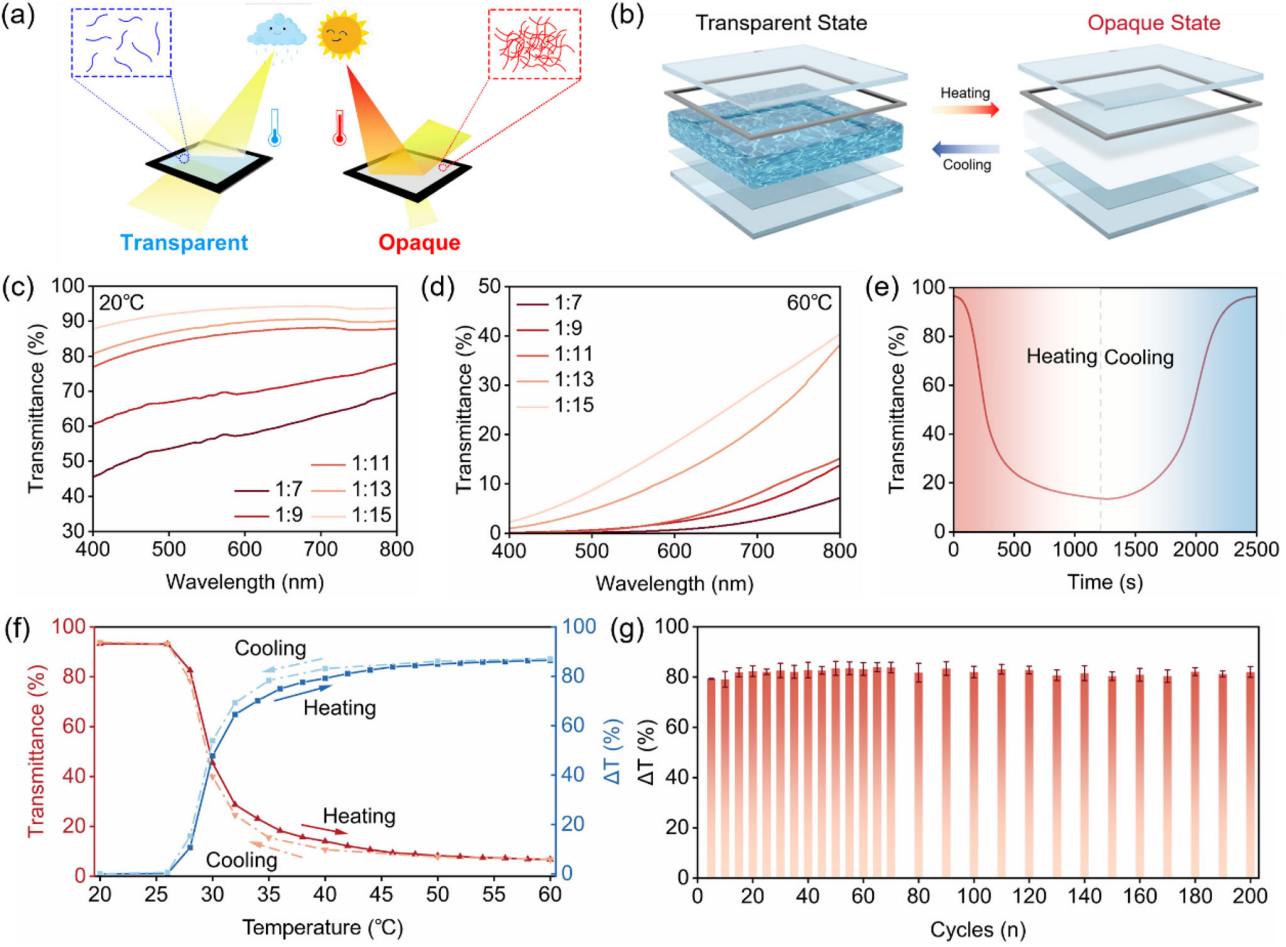
Evaluation of the hydroxyl cellulose‐based thermochromic hydrogels. (a) Schematic illustration of the TC mechanism of the HPC hydrogel electrolyte. (b) Diagram showing the TC behavior of the device. (c) Visible light transmittance spectra in the transparent and (d) opaque state for hydrogel electrolyte with different HPC: AM ratios. (e) Dynamic transmittance variation during heating and cooling cycles. (f) Transmittance vs. heating‐cooling temperature. (g) Transmittance modulation over 200 cycles.

The temperature‐responsive HPC hydrogel electrolyte was firstly synthesized via a one‐pot method (see Methods section for details), where acrylamide (AM) was selected as the polymeric host with HPC incorporated (Figure S1). During polymerization, AM reacts with the HPC molecular chains to form a 3D network, further strengthened by strong hydrogen bonding between the amino groups of AM and the hydroxyl groups of cellulose [43]. Additionally, metal cations can interact strongly with the amino, hydroxyl, and carbonyl groups present in the network [44]. However, the excessively strong interaction between the hydroxyl groups of cellulose and metal ions (e.g., K^+^, Zn^2+^) may induce precipitation, and can affect the initial T% and TC behavior of the hydrogels [45]. The use of bimetallic salts comprising potassium (K^+^) and zinc (Zn^2+^) ions in the electrolyte aim to accomplish the EC behavior in the PH window (see inset in Figure 1a) through the K^+^ (de)intercalation from/into the Prussian Blue electrode, with Zn^2+^ ions balancing the charge on the counter electrode.

In order to minimize interactions between metal ions and hydroxyl groups, HPC hydrogel was first synthesized to allow preferential crosslinking between hydroxyl groups of HPC and AM monomers, thus greatly reducing the number of exposed hydroxyl groups. Subsequent immersion in metal salt solution enables cations to interact with the remaining hydroxyl sites in a controlled manner, facilitating orderly ion alignment and transport necessary for EC process. Such a stepped polymerization‐immersion allows fast ion migration in the HPC hydrogel electrolyte without affecting thermochromism. However, excessively high cellulose content can still lead to low T% after metal ion uptake. Therefore, hydrogels with different HPC: AM ratios (1: 7, 1: 9, 1: 11, 1: 13, and 1: 15) were formulated and evaluated in subsequent discussions.

Transmittance spectra of the hydrogel electrolyte in both the transparent (low‐temperature) and opaque (high‐temperature) states were measured across the VIS range, indicating that decreasing the HPC content can increase the T% at both the clear and opaque states (Figure 2c, d). Lower HPC loadings can minimize the interaction between hydroxyl groups and K^+^/Zn^2+^, ensuring higher T% at the transparent state, yet the TC behavior at elevated temperature is also mitigated. HPC hydrogel electrolyte with HPC: AM ratio of 1: 11 exhibits the highest ΔT of ∼ 83.5% at 633 nm. Therefore, this optimal ratio was selected for all subsequent experiments unless otherwise stated.

The surface morphology of the HPC hydrogel examined by scanning electron microscopy (SEM) reveals an interconnected porous network (Figure S2a), providing abundant ion transport channels and facilitating K^+^/Zn^2+^ migration within the electrolyte. The cross‐sectional SEM image (Figure S2b) further indicates a relatively large pore size, which is conducive to efficient ion diffusion through the 3D matrix. Energy‐dispersive x‐ray spectroscopy (EDS) mapping confirms the successful incorporation of K^+^/Zn^2+^. Fourier‐transform infrared (FTIR) spectroscopy of the HPC hydrogel (Figure S3a) shows a broad and intense absorption band around 3360 cm^−1^, assigned to O─H stretching from cellulose, N─H stretching from AM, and extensive hydrogen bonding. Characteristic peaks at 1633, 1417, 1347, and 1112 cm^−1^ correspond to C═O stretching, C─H wagging, C─O stretching, and C‐C bending vibrations, respectively [46, 47]. Complementary Raman spectroscopy (Figure S3b) further elucidates the structural features, where the sharp peak at 3425 cm^−1^ arises from hydrogen‐bonded water molecules, absorption at 3256 cm^−1^ is associated with hydroxyl and amino groups and their hydrogen bonds, and peaks at 2931, 1660, 1433, 1327, 1108, and 939 cm^−1^ are attributed to ─CH_3_ symmetric stretching, C═O stretching, C─C stretching, C─O stretching, symmetric stretching of the C─O─C glycosidic bond, and in‐plane symmetric stretching of C─O─C, respectively [48, 49]. These spectroscopy analyses confirmed the successful crosslinking between HPC and AM to form HPC hydrogel electrolyte.

X‐ray photoelectron spectroscopy (XPS) was also conducted to analyze the chemical composition and bonding states (Figure S4). The C 1s spectrum is deconvoluted into four peaks at 284.2 eV (C═C), 284.8 eV (C─C), 285.9 eV (C─O), and 287.8 eV (C═O). The single peak at 399.2 eV in the N 1s spectrum is attributed to the C─N bonds in AM. Two main peaks at 531.0 eV (C─O) and 532.1 eV (hydroxyl groups from cellulose) are also observed in the O 1s spectrum [50, 51, 52]. The high‐resolution K 2p spectrum displays peaks at 292.4 and 295.2 eV, while the Zn 2p spectrum shows peaks at 1021.6 and 1044.5 eV, confirming the presence of K^+^/Zn^2+^ [53, 54].

Mechanical properties are critical for hydrogel electrolytes, especially for the potential application in flexible devices. Tensile tests conducted on hydrogels with different HPC: AM ratios show that increasing the cellulose content generally enhances both stress tolerance and strain capacity (Figure S5a). This improvement is attributed to additional crosslinking between HPC and the PAM network, as well as strong hydrogen bonding between hydroxyl and amino groups. Notably, the hydrogel with a 1: 11 HPC:AM ratio demonstrates excellent stretchability, with a fracture strain exceeding 500% (Figure S5b). Finally, the ionic conductivity of the HPC hydrogel electrolyte was assessed under various deformations using a universal testing machine (Figure S6). After 1500 cycles of stretching, bending, and twisting, the ionic conductivity increased by 6.7%, ∼247.7%, and ∼201.61%, respectively. This enhancement is attributed to the expansion of the hydrogel's network channels during these deformations. In contrast, 1500 compression cycles led to a ∼67.68% decrease in conductivity, resulting from the collapse and damage of the porous network structure. These mechanical tests highlighted the excellent deformability of the HPC hydrogel electrolyte needed for the assembly of durable PH windows.

Followingly, the reversible TC behavior was confirmed by cyclic heating and cooling tests (Figure 2e). At 60°C, the T% at 633 nm dropped from 96.5% to 13.5% within 1200 s and fully recovered to 96.5% after 1300 s of cooling, showing large and reversible T% modulation. The LCST of the HPC hydrogel is defined as the temperature corresponding to an optical contrast (ΔT) of 50%. Calculations indicate that the LCST of the HPC hydrogel (1: 11 HPC:AM ratio) is approximately 30°C (Figure 2f). This value is lower than several previously developed TC devices [16, 23, 32, 39, 41], demonstrating its promising application potential in diverse fields. Furthermore, the hydrogel demonstrated excellent optical stability over more than 200 heating–cooling cycles without significant performance degradation (Figure 2g), underscoring its robustness for long‐term operation.

## 5 Electrochemical and Electrochromic Performance of the PB/HPC System

Inspired by the successful formulation of ion‐conductive TC hydrogel electrolyte, EC electrode was subsequently prepared. Prussian blue (PB) electrode on FTO glass was synthesized via hydrothermal method, ensuring good adhesion, structural integrity, and uniformly distributed PB nanoparticles (Figure S7a). The x‐ray diffraction (XRD) pattern (Figure S7b) confirms the successful crystallization of PB, with all peaks corresponding to the Fe_4_[Fe(CN)_6_]_3_ phase (face‐centered cubic, PDF# 01‐0239), without impurities detected. The successful preparation of PB electrode was further corroborated by XPS analysis. The C 1s spectrum (Figure S8a) is fitted with peaks at 284.8 eV (C─C), 285.1 eV (C≡N), and 288.8 eV (C═O). The N 1s spectrum (Figure S8b) shows two main peaks at 397.7 and 398.9 eV, corresponding to cyanide nitrogen coordinated to Fe^2+^ and Fe^3+^, respectively. The Fe 2p spectrum (Figure S8c) exhibits characteristic peaks at 708.6 eV [Fe 2p_3/2_ (Fe^2+^)], 709.05 eV [Fe 2p_3/2_ (Fe^3+^)], 721.85 eV [Fe 2p_1/2_ (Fe^3+^)] and 721.36 eV [Fe 2p_1/2_ (Fe^2+^)], consistent with the Fe_4_[Fe(CN)_6_]_3_ structure, along with satellite peaks for Fe^3+^ and Fe^2+^ at 712.79 eV and 724.58 eV [55, 56, 57].

Electrochemical and EC behavior of the PB electrode was investigated using a two‐electrode configuration (Figure 3a), where the PB electrode was laminated with the HPC hydrogel electrolyte, with Zn foil as counter/reference electrode. During discharge, K^+^ intercalates into the PB lattice, reducing Fe^3+^ to Fe^2+^ and bleaching the electrode to a transparent state. During charging, K^+^ de‐intercalates from the PB lattice, oxidizing Fe^2+^ to Fe^3+^ and causing the PB coloration [38]. XPS analysis corroborates this process, as illustrated in Figure S9, where the peak area ratio of Fe^3+^ to Fe^2+^ in oxidized PB is substantially larger than that in the reduced state. The corresponding cyclic voltammetry (CV) curves at a scan rate of 20 mV s^−1^ (Figure 3b) exhibit a well‐defined pair of redox peaks, confirming the reversible redox reaction of the PB electrode. Furthermore, the CV measurements at 60°C (Figure S10) reveals narrowed voltage polarization and comparable current response with those at room temperature, indicating increased electrochemical activity of PB brought by the TC HPC hydrogel electrolytes after phase transition.

**FIGURE 3:**
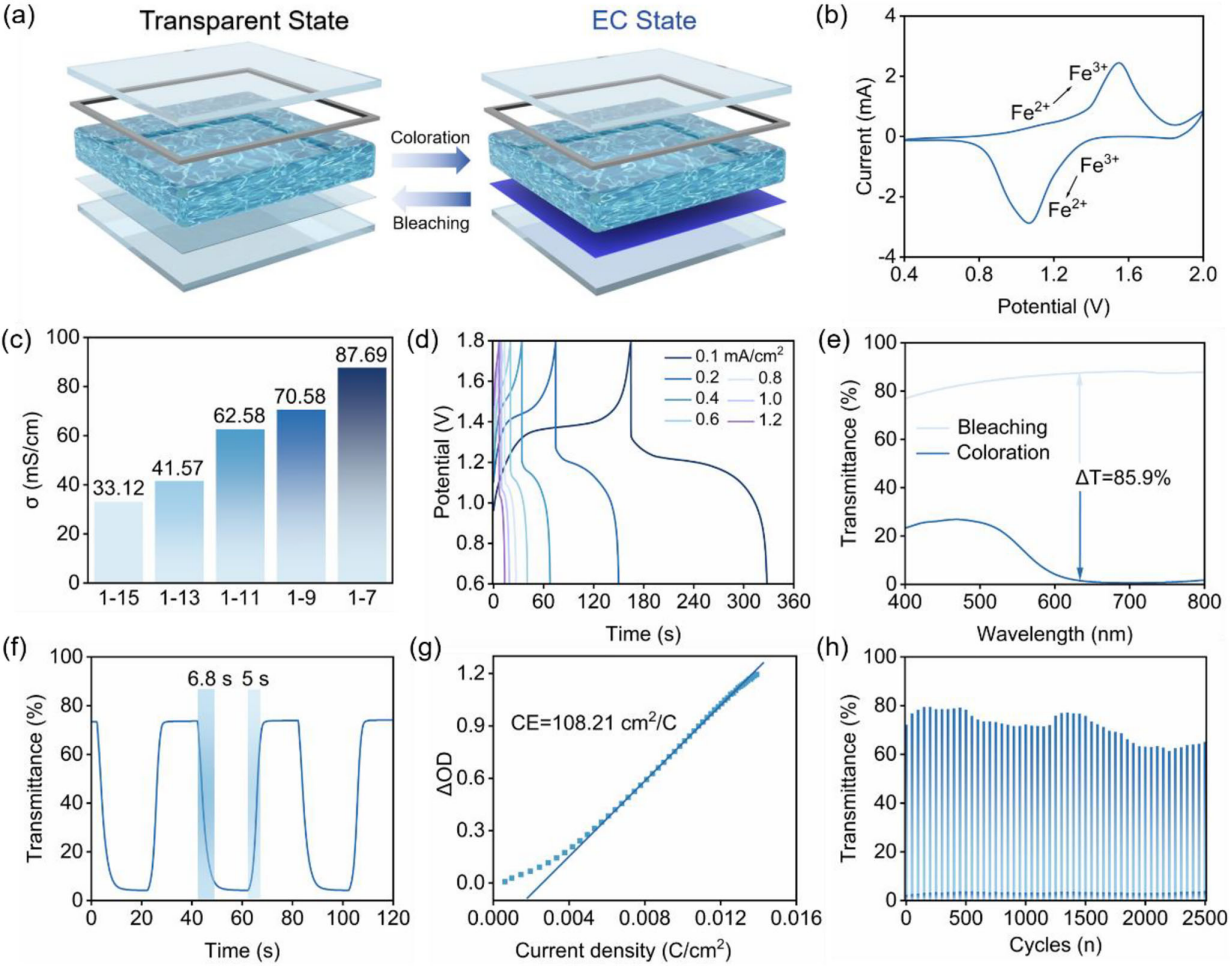
Electrochemical and electrochromic performance of Prussian blue electrode in the hydroxyl cellulose‐based hydrogel electrolyte. (a) Schematic illustration of the EC mechanism of the Prussian blue (PB) electrode. (b) Cyclic voltammetry (CV) curve at a scan rate of 20 mV s^−1^ and (c) ionic conductivity of HPC hydrogel electrolyte with different ratios. (d) Galvanostatic charge–discharge (GCD) profiles at different current densities. (e) VIS transmittance spectra in the bleached and colored states. (f) Switching times for coloration and bleaching. (g) Coloration efficiency. (h) Optical modulation stability over 2500 cycles.

To quantify the ionic diffusion coefficient in PB electrode, galvanostatic intermittent titration technique (GITT) was utilized during charge and discharge cycles (Equation 1),

(1) $$D=\frac{4}{\pi \tau }{\left(\frac{m_{B}V_{B}}{M_{B}S}\right)}^{2}{\left(\frac{\Delta E_{S}}{\Delta E_{\tau }}\right)}^{2}$$

where τ denotes the current pulse duration, V_B_ and M_B_ correspond to the molar volume and molar mass of the electrode material, respectively. m_B_ represents the mass of active electrode material, and S is the geometric contact area of the electrode. ΔE_S_ stands for the steady‐state voltage variation, while ΔE_τ_ refers to the total voltage shift generated during the current pulse. The results indicate that the diffusion coefficient fluctuations in the PB electrode are highly symmetric during galvanostatic charge and discharge processes. The average ionic diffusion coefficients are calculated to be 2.50351 × 10^−9^ (cm^2^ s^−1^) for charging and 4.24838 × 10^−9^ (cm^2^ s^−1^) for discharging, respectively (Figure S11a). The comparable diffusion coefficients during charge and discharge further accounts for the outstanding cycling stability of PB electrodes. The relatively high diffusion coefficient, particularly for reduction, can be attributed to the small ionic radius of K^+^, which facilitates rapid (de)intercalation within the PB electrode framework. Furthermore, the relationship between the peak current I and the scan rate v is analyzed to unveil the charge storage mechanism (Equation 2).

(2) $$I=av^{b}$$

A value of b = 0.5 indicates a diffusion‐controlled electrochemical process, while b = 1 suggests a surface‐capacitive process. The calculated b values for the oxidation and reduction processes of the PB electrode are 0.63 and 0.7 (Figure S11b), respectively, indicating the dominant diffusion‐controlled redox reaction. The electrochemical durability of PB electrode in the HPC hydrogel electrolyte was further assessed by continuous CV scans (Figure S12), showing negligible shape changes after 1000 cycles, demonstrating excellent electrochemical reversibility. Minor peak shifts are attributed to polarization effects during repeated K^+^/Zn^2+^ (de)intercalation.

Using electrochemical impedance spectroscopy (EIS) Nyquist plots (Figure 3c and Figure S13), the intrinsic ionic conductivity of the HPC hydrogel electrolytes with HPC: AM ratios of 1: 7, 1: 9, 1: 11, 1: 13, and 1: 15 were determined to be 87.69, 70.58, 62.58, 41.57, and 33.12 mS cm^−1^, respectively, while a pure polyacrylamide (PAM) hydrogel without HPC exhibited a significantly lower conductivity of 12.77 mS cm^−1^ (Figure S14). The hydrogel electrolyte in this work with a 1: 11 molar ratio exhibits an ionic conductivity that also outperforms those reported in numerous excellent previously published studies [58, 59, 60, 61, 62, 63]. The enhanced conductivity with increasing HPC content is ascribed to the greater number of exposed hydroxyl groups within the hydrogel network, which facilitates more ordered ion transport. The optimized 1: 11 HPC hydrogel also shows a steadily increasing ionic conductivity with temperature (Figure S15), corroborating the smaller voltage polarization in the CV scans of PB at 60°C (Figure S10), likely due to facilitated ion migration at higher temperatures, thereby optimizing the electrochemical performance [64]. Additionally, the Zn^2+^ transference number for the 1: 11 HPC hydrogel is 0.322, higher than that of the pure PAM hydrogel (Figure S16). This suggests a transition from a disordered, predominantly 2D diffusion pathway in PAM to a more stable 3D ion transport network in the HPC‐based gel, which is beneficial for suppressing zinc dendrite formation [65, 66].

Galvanostatic charge–discharge (GCD) tests of PB in HPC hydrogel electrolyte reveal its promising energy storage capability (Figure 3d). The GCD curves at different current densities are nearly symmetrical, indicating high Coulombic efficiency and good reversibility. The calculated areal capacities at current densities from 0.1 to 1.2 mA cm^−2^ are 45.3, 41.9, 37.7, 33.8, 30.0, 26.4, and 22.0 mAh m^−2^. This high areal capacity stems from the abundant nanoparticles in the PB electrode and the efficient ion transport channels provided by the hydroxyl‐rich HPC hydrogel network, outperforming many reported zinc‐ion batteries and highlighting its potential for integrated energy storage [67, 68, 69, 70, 71]. An as‐assembled proof‐of‐concept device (5 × 5 cm^2^) successfully powered a digital hygrothermograph for nearly 9 h (Figure S17), demonstrating its practical utility in dual‐functional EC‐energy storage applications.

The excellent electrochemical performance of PB electrode projected its excellent EC behavior. The transmittance spectra of PB electrode at bleached (0.6 V) and colored (1.8 V) states were recorded across the VIS range, reaching a remarkable transmittance modulation (ΔT) of 85.9% at 633 nm (Figure 3e). Dynamic transmittance variation profiles also demonstrate fast switching kinetics with a coloration time of 6.8 s and a bleaching time of 5.0 s (Figure 3f), and a high coloration efficiency (CE) of 108.21 cm^2^ C^−1^ (Figure 3g), outperforming PB electrode in counterpart PAM hydrogel electrolytes (CE = 93.34 cm^2^ C^−1^, Figure S18). An excellent long‐term optical stability was also achieved, with ΔT retention of 77% after 2500 coloring/bleaching cycles (Figure 3h). The excellent cycling stability was verified by the structural integrity of the cycled PB electrode (Figure S19).

Finally, to verify the unique merits of the dual‐cation electrolyte, the switching kinetics pf PB electrode in K^+^‐only HPC hydrogel electrolytes was also evaluated via both two‐electrode and three‐electrode configurations (Figure S20a). Compared to Figure 3f and Figure 3h, in the absence of Zn^2+^, the PB electrode showed obvious extended response times and significantly sacrificed long‐term stability (Figure S20), primarily due to the charge imbalance on the Zn anode. Nonetheless, in three‐electrode testing, PB still shows comparable switching kinetics in K^+^‐only HPC hydrogel electrolytes. These results suggest the advantages and necessity in formulating K^+^‐Zn^2+^ dual‐cation electrolytes to allow the assembly of fast‐switching and durable electrochromic devices, and indicates the dominant K^+^ (de‐)intercalation induced PB coloration/bleaching.

## 6 Energy‐Saving Performance of the PH Smart Window

By utilizing electrical fields, temperature changes, or a combination of both, four distinct operating modes were achieved (Figure 4a) in the assembled PH smart window, enabling flexible regulation of indoor temperature and window appearance. When the ambient temperature is below the LCST and the PB electrode is electrochemically bleached, the window remains highly transparent, allowing sunlight to enter the room. Once the outdoor temperature exceeds the LCST, the window autonomously switches to a TC state with a milky appearance. This highly scattering state effectively blocks sunlight and heat, helping to maintain a cooler and more comfortable indoor environment. During hot summer, the heat‐blocking capability can be further enhanced by electrically switching the PB film to its blue‐colored state. The superimposed EC‐TC effects result in a mode with extremely low light T%, significantly reducing energy consumption while ensuring indoor privacy. Even in cold winters when the ambient temperature remains below the LCST, a certain degree of shading can still be achieved by activating the EC coloring of the PB electrode when needed. In short, the innovative PH smart window responds intelligently to both electrical and thermal stimuli, enabling tailored indoor lighting and thermal management according to varying environmental conditions.

**FIGURE 4:**
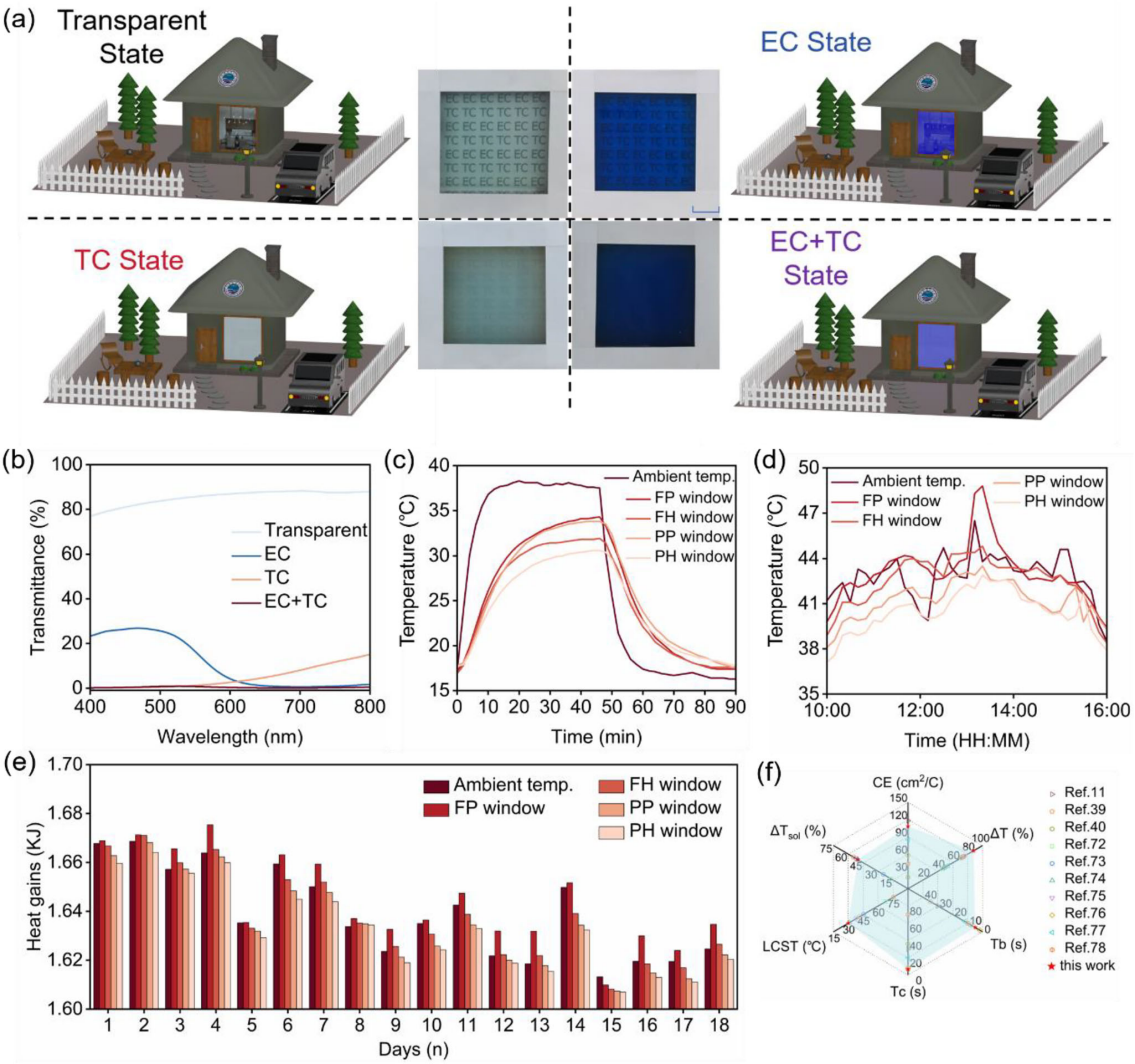
Optothermal regulation performance of the dual‐responsive smart window. (a) Schematics and photographs of the PH smart window under four operating states, scalebar 1cm. (b) VIS transmittance spectra of PH cuvette at the four operating states. (c) Indoor temperature variations under infrared lamp illumination. (d) Internal temperature variations of house models installed with different windows during a typical sunny day. (e) Changes in heat gain under outdoor simulation with multiple consecutive sunny days. (f) Comparison of the key performance parameters.

The transmittance spectra of the EC‐TC coupled modulation were measured in the four operational states, in a PH cuvette set‐up. As shown in Figure 4b, PH cuvette in EC state exhibits low T% in the 600–800 nm range but higher T% between 400–600 nm. Comparatively, PH cuvette in TC state shows low T% at 400–600 nm and higher T% at 600–800 nm. Remarkably, the coupled EC‐TC state ensures an extremely low T% (< 0.9%) across the VIS spectrum (400–800 nm), demonstrating exceptional optical modulation capability of the dual‐responsive PH cuvette. Furthermore, the cycling stability of the PH smart window under coupled EC+TC switching was also tested (Figure S21), showing excellent durability after 200 consecutive EC+TC cycling, demonstrating promising long‐term operational stability for practical applications.

To evaluate the energy‐saving performance of the PH smart window, a miniature house model (Figure S22) was constructed to simulate real‐world conditions, with internal dimensions of 24.5 × 12.7 × 14.5 cm^3^. The interior was lined with black light‐blocking polyethylene terephthalate (PET), and the exterior was wrapped with aluminum foil. A 5 × 5 cm^2^ square opening was made on the top for window installation, with an active area of 3 × 3 cm^2^. Four types of windows were prepared for comparison, a conventional window containing only polyacrylamide (PAM) and FTO glasses (labeled as FP), a TC smart window with HPC and FTO (FH), an EC smart window with PAM and PB (PP), and the coupled EC‐TC smart window with HPC and PB (PH).

Indoor energy‐saving performance was evaluated using an infrared lamp as a light and heat source, placed 25 cm above the window and operating at its maximum power of 275 W. As shown in Figure 4c, the coupled PH smart window exhibited excellent thermal insulation. With an effective area of only 3 × 3 cm^2^, the internal house temperature was reduced by approximately 7°C compared to the ambient temperature after 50 min of infrared lamp irradiation, and by 3.7°C, 1.5°C, and 3.2°C compared to the FP, FH, and PP windows, respectively. Furthermore, under the shorter heating duration of 20 min, the dual‐responsive PH smart windows still ensured an internal temperature 8.6°C lower than the ambient, 4.1°C lower than the FP window, 1.0°C lower than the FH smart window, and 1.2°C lower than the PP smart window (Figure S23). Moreover, the PH window showed stable heat‐regulation performance over ten heating‐cooling cycles (infrared lamp irradiation for 20 min, and cooling at room temperature for 40 min in each cycle), maintaining a comfortable internal temperature of ∼ 28°C (Figure S24).

In addition to indoor testing, outdoor testbeds were also fabricated and monitored. The ambient temperature and the internal temperature data in the house models were recorded continuously from 8:00 AM to 7:00 PM (Figure S25). As shown in Figure 4d, the internal temperature of the PH smart window house remained consistently lower than that of the FP window, the FH thermochromic window, and the PP electrochromic window. Notably, during hot daytime hours, the PH window maintained a temperature significantly lower than the ambient, demonstrating its potential to reduce building cooling loads. Furthermore, stability tests were conducted over several consecutive sunny days (Figure 4e), confirming that the PH smart window experienced the lowest heat gain, underscoring its superior and reliable thermal insulation performance.

In short, the outstanding thermal insulation and light modulation capabilities of the PH smart window demonstrate its significant potential for energy‐saving applications, outperforming those reported in literature in key performance parameters (Table S1 and Figure 4f) [11, 39, 40, 72, 73, 74, 75, 76, 77, 78, 79].

## 7 Global Energy‐Saving Simulation of the PH Smart Window

Given the superior optothermal regulation performance of the PH smart window, its building energy modulation potential was thereby simulated. A medium‐sized office building model integrated with the PH smart window was constructed using OpenStudio, and EnergyPlus was employed to simulate its energy‐saving potential across diverse climatic conditions. While practical factors such as device degradation and voltage decay would affect real‐world performance, these simulations provide a theoretical estimate of the energy‐saving capability.

Taking Shenzhen (China) in the Northern Hemisphere as an example, the annual energy‐saving performance of the dynamic window was analyzed based on local climate data and the optical properties of the PH smart window (Table S2). As shown in Figure 5a, compared to a conventional static window, the PH smart window in either EC, TC or EC+TC modes can reduce energy demand in every month, achieving an annual total energy saving of approximately 31%–41%. Similarly, using Rio de Janeiro (Brazil) as a representative case in the Southern Hemisphere (Figure 5b), the PH smart window also demonstrated reduced monthly energy demand across all months compared to a conventional window, with an annual energy saving of about 36%–45%.

**FIGURE 5:**
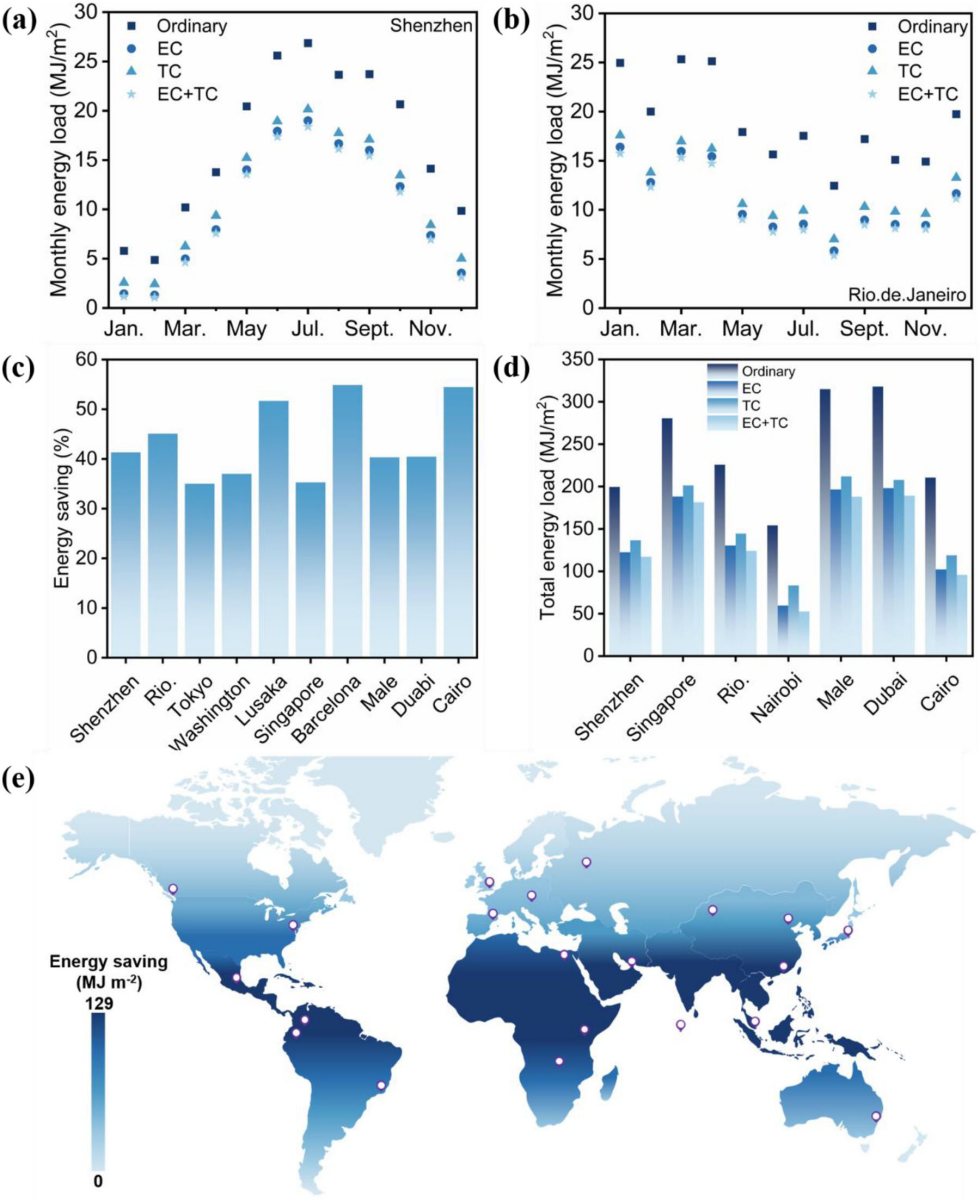
Building energy consumption simulation and calculation. Annual energy load calculation for a conventional window and the PH smart window in different states in (a) Shenzhen (China), and (b) Rio de Janeiro (Brazil). (c) Energy‐saving percentages of the PH smart window compared to an ordinary window across ten different regions. (d) The calculated total annual building energy consumption for seven different regions worldwide. (e) Simulated global energy‐saving performance of the PH smart window.

The energy‐saving effect varies significantly across different latitudes due to distinct heating and cooling demands. In low‐latitude regions with high solar intensity for most of the year, energy consumption is dominated by cooling, and the dynamic window exhibits more pronounced energy savings. Calculations of the annual total energy saving relative to ordinary windows across ten representative global regions (Figure 5c) reveal that the smart window can reduce annual energy consumption by 35% to 55%. Furthermore, numerical evaluations of the annual total energy demand of dynamic windows across 7 representative global regions (Figure 5d) further corroborated that smart windows offer a marked reduction in energy consumption compared with ordinary window systems. Finally, the energy‐saving performance of the PH smart window was evaluated across 22 major global cities. The results confirm the excellent energy efficacy of our PH smart window, particularly in tropical regions (Figure 5e), where it demonstrates the potential to save up to 129 MJ m^−2^ in energy consumption, equivalent to reducing CO_2_ emissions by approximately 505.68 kg m^−2^.

## 8 Conclusion

Given the limited T% modulation of ion insertion/extraction‐based EC windows in the VIS range and hydrogel‐based TC windows in the NIR range, we have herein developed a dual‐responsive electro‐thermochromic PH smart window by integrating an EC PB electrode with a TC HPC hydrogel electrolyte, achieving large T% modulation across the solar spectrum. With optimized HPC: AM ratio, the hydrogel electrolyte offers high ionic conductivity (62.58 mS cm^−1^) at room temperature, excellent mechanical stability (tensile strain > 500%), and excellent TC performance with a low LCST of 30°C, thereby ensuring excellent optothermal regulation properties in the as‐assembled dual‐responsive PH smart window. This PH smart window demonstrates remarkable optical modulation (ΔT_lum_ = 87.3%, ΔT_sol_ = 50.5%), fast EC switching speeds (6.8 s for coloration and 5.0 s for bleaching), a high CE of 108.21 cm^2^ C^−1^, and outstanding cycling stability (over 2500 cycles for the EC PB electrode and over 200 cycles for the TC HPC hydrogel electrolyte). The PH window can reversibly and rapidly switch among four distinct operating modes in response to electrical and thermal stimuli, exhibiting superior thermal management capabilities, reducing the indoor temperature by up to 8.6°C under infrared lamp illumination, and achieving significant annual building energy savings of up to 129 MJ m^−2^. This innovative dual‐functional smart window advances the development of multifunctional platforms and holds strong potential for practical applications in building energy modulation.

## 9 Experimental Section

### 9.1 Materials

Hydroxypropyl cellulose (HPC, M_w_ ≈ 100,000), acrylamide (AM), N,N'‐methylenebisacrylamide (MBA), tetramethylethylenediamine (TEMED), potassium ferricyanide (K_3_[Fe(CN)_6_]), glucose, and zinc acetate (ZnAc_2_) were purchased from Aladdin. Ammonium persulfate (APS) and potassium chloride (KCl) were obtained from Macklin. Hydrochloric acid (HCl) was supplied by Shanghai Hushi. Fluorine‐doped tin oxide (FTO) coated glass substrates were purchased from Wuhan Jingge.

### 9.2 Fabrication of the Prussian Blue Electrode

Glucose (0.25 g) and K_3_[Fe(CN)_6_] (0.66 g) were dissolved in deionized (DI) water (60 mL), followed by adding HCl (1 mL, 36 – 38 wt.%) into the mixed solution. FTO glass substrates were ultrasonically cleaned in DI water and ethanol, respectively, and then air‐dried. The cleaned FTO glass was placed in a Teflon‐lined autoclave with the conductive side facing downward. The prepared precursor solution was poured into the autoclave, fully immersing the FTO glass. After the autoclave was heated at 120°C for 4 h, the FTO glass was taken out, thoroughly rinsed with DI water, and dried at 60°C overnight to obtain the PB electrode.

### 9.3 Synthesis of the HPC Hydrogel

First, HPC (0.125 g) was dissolved in DI water (15 mL) under vigorous stirring, followed by sonication for 30 min. Then, AM (1.375 g), MBA (75 mg), and APS (405 mg) were added to the HPC solution and stirred until fully dissolved. Subsequently, TEMED (15 µL) was added dropwise to the mixture with gentle stirring, and the resulting solution was quickly poured into a custom‐made mold, which consisted of two 10 × 10 cm^2^ glass plates separated by a 3‐mm‐thick square silicone spacer. Gelation was carried out at 60°C for 90 min. Finally, the as‐prepared hydrogel was immersed in a mixed aqueous solution containing KCl (1 M) and ZnAc_2_ (0.1 m) for 12 h to obtain the HPC hydrogel electrolyte.

### 9.4 Assembly of the PH Smart Window

The HPC hydrogel electrolyte was placed onto the PB electrode. A zinc foil and a piece of FTO glass (conductive side facing inward) were sequentially stacked on top of the hydrogel to form a sandwich structure. The edges of the assembled device were sealed to complete the fabrication of the PH smart window.

### 9.5 Material Characterization

The microstructure and morphology of the PB electrode and the HPC hydrogel were examined using a field‐emission scanning electron microscope (Gemini SEM 300). Grazing‐incidence x‐ray diffraction (GIXRD) patterns were collected on a Rigaku SmartLab diffractometer. X‐ray photoelectron spectroscopy (XPS) analysis was performed on a Thermo Scientific K‐Alpha spectrometer. Raman spectra were acquired using a Horiba LabRAM HR Evolution spectrometer with a 1064 nm laser excitation source. Fourier‐transform infrared (FTIR) spectroscopy was conducted on a Thermo Fisher Scientific Nicolet iS20 spectrometer.

### 9.6 Electrochemical and Device Performance Tests

All electrochemical measurements were performed using a CHI760E electrochemical workstation (CH Instruments, China) in a two‐electrode configuration, where the PB electrode served as the working electrode and the zinc foil as the counter/reference electrode. Cyclic voltammetry (CV), chronoamperometry (CA), and galvanostatic charge–discharge (GCD) tests were conducted under ambient conditions. Electrochemical impedance spectroscopy (EIS) measurements were carried out in the frequency range of 0.01 Hz to 100 kHz. The ionic conductivity (σ) of the hydrogel was calculated using the formula (Equation 3).

(3) $$\sigma =\frac{L}{A\times R}$$

where L is the thickness of the sample (cm), A is the effective contact area between the electrode and the electrolyte (cm^2^), and R is the intrinsic bulk resistance (Ω) obtained from the intercept of the EIS Nyquist plot on the real axis.

Optical performance was characterized in both ex situ and in situ modes using a Shimadzu UV‐2600i spectrophotometer coupled with a custom‐built temperature‐controlled stage. Coloration efficiency (CE) was calculated according to the following formula (Equation 4):

(4) $$CE=\frac{\Delta OD}{Q_{A}}=\frac{log\frac{T_{b}}{T_{c}}}{Q/A}$$

where Q_A_ is the areal charge density (C cm^−2^), T_b_ and T_c_ are the transmittances (%) in the bleached and colored states at a specific wavelength, respectively, Q is the total charge (C) passed through the electrode, and A is the effective electroactive area of the electrode (cm^2^). These parameters are used for calculating the coloration efficiency of the electrochromic device.

Galvanostatic intermittent titration technique was measured on the Neware battery tester.

### 9.7 Energy‐Saving Simulation

The quantification of heat gains was achieved by calculating the thermal load, defined as the energy required to heat the air volume inside the test enclosure, based on the following formula (Equation 5):

(5) Q=m×c×dT

where Q is the heat load, m is the mass of air, C is the specific heat capacity of air, and dT is the average temperature difference between real temperature recorded inside the testbed and the targeted indoor temperature.

The optical performance of the device was characterized using a UV–vis–NIR spectrophotometer (Shimadzu UV‐3600). For the global energy‐saving simulation, the luminous transmittance (T_vis_, 380–780 nm), near‐infrared transmittance (T_inf_, 1500–2500 nm), and total solar transmittance (T_sol_, 300–2500 nm) were calculated using the following formula (Equation 6):

(6) $$T_{sol/vis/inf}=\frac{\int \varphi_{sol/vis/\inf}\left(\lambda \right)T\left(\lambda \right)d\lambda }{\int \varphi_{sol/vis/\inf}\left(\lambda \right)d\lambda }$$

where T(λ) represents the transmittance at a specific wavelength λ, φ_vis_(λ) is the standard luminosity function for photopic vision, and φ_sol_(λ) denotes the solar irradiance spectrum under Air Mass 1.5 (AM 1.5) conditions. The corresponding modulation in transmittance can be calculated by taking the difference between the values in different states.

Similarly, the reflectance in the visible range (380–780 nm, R_vis_), in the infrared range (1500–2500 nm, R_inf_), and in the full solar spectrum (300–2500 nm, R_sol_) are calculated using the following formula (Equation 7):

(7) $$R_{sol/vis/inf}=\frac{\int \varphi_{sol/vis/\inf}\left(\lambda \right)R\left(\lambda \right)d\lambda }{\int \varphi_{sol/vis/\inf}\left(\lambda \right)d\lambda }$$

where R(λ) represents the reflectivity at a specific wavelength λ, φ_vis_(λ) is the standard luminosity function for photopic vision, and φ_sol_(λ) is the solar radiation spectrum under Air Mass 1.5 conditions.

According to Kirchhoff's law, the spectral emissivity (ε_NIR_) equals the spectral absorptivity (α_NIR_). Since the smart window is essentially opaque in the infrared band, considering its low transmittance (T%) within this wavelength range, its spectral emissivity in the NIR band can be obtained via ε_NIR_ = α_NIR_ = 1−R_NIR_. The annual total heating and cooling energy consumption E (kWh m^−2^) for an office building should be calculated in accordance with the following regulation (Equation 8):

(8) $$E=E_{H}+E_{C}$$

E_H_ represents the annual heating energy consumption (kWh m^−2^). E_C_ denotes the annual cooling energy consumption (kWh m^−2^). The annual heating energy consumption for buildings in the following climate zones—Hot Summer and Warm Winter, Hot Summer and Cold Winter, and Temperate zones—should be calculated according to the following equation (Equation 9):

(9) $$E_{H}=\frac{Q_{H}}{A\times COP_{H}}$$

where Q_H_ is the cumulative annual heating demand (kWh) obtained through dynamic simulation software, A is the total floor area (m^2^), and COP_H_ is the coefficient of performance of the heating system, taken as 2.6.

The annual cooling energy consumption should be calculated according to the following equation (Equation 10):

(10) $$E_{C}=\frac{Q_{C}}{A\times COP_{C}}$$

where Q_C_ is the cumulative annual cooling demand (kWh) obtained through dynamic simulation software, A is the total floor area (m^2^), and COP_C_ is the coefficient of performance of the cooling system. For public buildings, COP_C_ is taken as 3.5; for residential buildings in the Cold region, Hot Summer and Cold Winter zone, and Hot Summer and Warm Winter zone, COP_C_ is taken as 3.6.

The actual energy savings should be calculated according to the following equation (Equation 11):

(11) $$E_{A}=E_{G}-E_{i}$$

where E_A_ is the actual net energy saving, E_G_ is the total gross energy saving achieved by the PH smart window intervention, and E_i_ is the energy consumed by the operation of the PH smart window itself. Notably, the annual energy consumption induced by smart window switching (E_i_) is negligible compared to the energy saving achieved, therefore E_i_ was neglected for energy saving simulations.

Based on weather files from the EnergyPlus database, we simulated the building energy consumption for 22 cities worldwide, including Beijing, Shenzhen, Ürümqi, Tokyo, Singapore, Vancouver, Santiago, Bogotá, Barcelona, London, Vienna, Lusaka, Malé, Dubai, Moscow, Giza, Nairobi, Cairo, Rio de Janeiro, Washington, D.C., Mexico City, and Sydney.

## Author Contributions

**Tongyu Chen**: methodology, software, data curation, writing – original draft, formal analysis, investigation. **Bing Xu**: software, data curation, investigation, formal analysis. **Bingkun Huang**: software, data curation, formal analysis. **Guolong Zhou**: data curation, investigation, formal analysis. **Zhongyang Wang**: validation, visualization, resources. **Pooi See Lee**: validation, visualization, resources. **Xiaohui Tang**: validation, visualization, resources. **Wu Zhang**: validation, visualization, writing – review and editing. **Jingwei Chen**: conceptualization, supervision, funding acquisition, visualization, validation, project administration, resources, writing – review and editing, methodology. **Wenting Wu**: software, investigation, data curation. **William W. Yu**: validation, visualization, resources.

## Conflicts of Interest

The authors declare no conflict of interest.

## Supporting information




**Supporting File**: advs77055‐sup‐0001‐SuppMat.docx.

## Data Availability

The data that support the findings of this study are available from the corresponding author upon reasonable request.
