# Supplementary material for: Advancing Energy Efficiency in Smart Windows via Dual‐Responsive Electro‐Thermochromism
Source: Adv Sci (Weinh). 2026 Aug 7;13(60):e77055. doi: 10.1002/advs.77055 (PMC13449530; doi:10.1002/advs.77055)
Supplement: Supplementary file 1 [file ADVS-13-e77055-s001.docx]

Supporting information

Advancing Energy Efficiency in Smart Windows via Dual-responsive Electro-Thermochromism

Tongyu Chen^1^, Bing Xu^1^, Guolong Zhou^1^, Wenting Wu^2^, Bingkun Huang^3,4^, Xiaohui Tang^5,^ *, William W. Yu^3, 4^, Pooi See Lee^2^, Wu Zhang^6,^ *, Zhongyang Wang^7,^ *, Jingwei Chen^1, 8,^ *

**Figure S1.** Schematic microstructure of the HPC hydrogel electrolyte.

**Figure S2.** SEM images of the (a) surface and (b) cross-section of the HPC hydrogel electrolyte, along with the corresponding energy-dispersive X-ray spectroscopy (EDS) mapping.

**Figure S3.** (a) Fourier-transform infrared and (b) Raman spectrum of the HPC hydrogel electrolyte.

**Figure S4.** Fitted XPS spectra of (a) C 1s, (b) N 1s, (c) O 1s, (d) K 2p and (e) Zn 2p in the HPC hydrogel electrolyte.

**Figure S5.** (a) Stress-strain curves of HPC hydrogel with different HPC:AM ratios and the bare PAM hydrogel. (b) Optical images of the 1:11 HPC hydrogel under tensile strain.

**Figure S6.** Changes in ionic conductivity of the HPC hydrogel electrolyte after 1500 cycles of repeated stretching, bending, twisting, and compression.

**Figure S7.** (a) SEM image and (b) XRD pattern of the as-prepared PB electrode.

**Figure S8.** Fitted XPS spectra of (a) C 1s, (b) N 1s and (c) Fe 2p in the PB electrode.

**Figure S9.** Fe XPS Spectra of PB electrodes in the (a) oxidation and (b) reduction states.

**Figure S10.** Comparison of CV Curves at ambient temperature and 60℃.

**Figure S11.** (a) GITT curves and corresponding diffusion coefficient distributions of PB electrodes during galvanostatic charge–discharge processes. (b) CV curves at different scan rates.

**Figure S12.** CV curves over 1000 cycles.

**Figure S13.** Nyquist plots of different HPC hydrogel electrolytes.

**Figure S14.** Comparison of ionic conductivity between the 1: 11 HPC hydrogel electrolyte and the pure PAM hydrogel electrolyte.

**Figure S15.** Temperature-dependent ionic conductivity of the 1: 11 HPC hydrogel electrolyte.

**Figure S16.** Current–time profiles for the 1: 11 HPC and the PAM hydrogel electrolyte.

**Figure S17.** Demonstration of the PH smart window powering a digital hygrothermograph over an extended period of nearly 9 hours.

**Figure S18.** Coloration efficiency of the PB electrode in PAM hydrogel electrolyte.

**Figure S19.** SEM image of the PB electrode after 2500 cycles.

**Figure S20.** (a) Response times of two-electrode and three-electrode systems, (b) cycling stability of the two-electrode system.

**Figure S21.** Cycling stability of PH smart windows under coupled EC+TC switching.

**Figure S22.** Schematic diagram of the miniature house model used for outdoor and indoor heat-blocking measurements.

**Figure S23.** Temperature changes inside the model house after 20 minutes of infrared lamp irradiation.

**Figure S24.** Indoor temperature variation of the dual-responsive PH smart window over 10 cycles under infrared irradiation.

**Figure S25.** Photograph of the outdoor energy-saving testing setups.

**Table S1.** Comparison between this work and other reported studies.

|  | **CE (C/cm^2^)** | **ΔT (%)** | **t_b_ (s)** | **t_c_ (s)** | **LCST (℃)** | **ΔT_sol_ (%)** | **Ref.** |
| --- | --- | --- | --- | --- | --- | --- | --- |
| AgNO_3_+CuCl_2_ | 118.1 | 72.5 | 30 | 30 | — | — | [1] |
| WO_3_ | 58 | 71.5 | 1.9 | 8.7 | — | — | [2] |
| TiO_2_ | 84.96 | 53.65 | 12 | 20 | — | — | [3] |
| VO_2_ | — | 45.7 | — | — | 67.3 | 16.9 | [4] |
| Tha_2_MAPbI_4_ | — | 48.5 | — | — | 45.3 | 24.7 | [5] |
| HPC | — | 71.6 | — | — | 31 | 50.3 | [6] |
| MC | — | 76 | — | — | 38 | 53 | [7] |
| WO_3_/HPC | — | 71.8 | 35 | 7 | 41 | 54.9 | [8] |
| WO_3_/VO_2_ | 19.92 | 50.02 | 8 | 36 | — | — | [9] |
| WO_3_/(nBAI + PbI_2_):L61 | 43.2 | 75.2 | 10 | 70 | 75.2 | — | [10] |
| **This work** | **108.21** | **87.3** | **5** | **6.8** | **36** | **50.5** |  |

**Table S2.** Key optical performance parameters of the PH smart window.

| **Parameters** | **Transparent** | **EC** | **TC** | **EC+TC** | **Static windows** |
| --- | --- | --- | --- | --- | --- |
| T_vis_ | 0.6370 | 0.0957 | 0.1122 | 0.0008 | 0.89 |
| T_sol_ | 0.550 | 0.107 | 0.1672 | 0.0454 | 0.84 |
| T_inf_ | 0.0113 | 0.0073 | 0.0114 | 0.0118 | 0 |
| Front-R_vis_ | 0.2751 | 0.2566 | 0.3142 | 0.2964 | 0 |
| Front-R_sol_ | 0.2129 | 0.2027 | 0.2423 | 0.2309 | 0 |
| Front-R_inf_ | 0.1625 | 0.1676 | 0.1673 | 0.1709 | 0.16 |
| Back-R_vis_ | 0.2771 | 0.2522 | 0.3073 | 0.2621 | 0 |
| Back-R_sol_ | 0.2123 | 0.1966 | 0.2351 | 0.2041 | 0 |
| Back-R_inf_ | 0.1498 | 0.1534 | 0.1537 | 0.1550 | 0.16 |
| ε_inf_ | 0.8262 | 0.8251 | 0.8213 | 0.8173 | 0.84 |
| Conductivity  (W (m·K)^-1^) | 1.4 | 1.4 | 1.4 | 1.4 | 0.9 |

**References**

[1]	Zhao, X.; Chen, Q.; Fan, F.; Wang, Q.; Su, Y.; Liu, M.*; et al.* "Dual-Band Electrochromic Smart Window for Dynamic Switching between Radiative Cooling and Solar Heating." *Advanced Science* 12 (2025): e04483, https://doi.org/10.1002/advs.202504483

[2]	Tong, X.; Wang, J.; Zhang, P.; Lei, P.; Gao, Y.; Ren, R.*; et al.* "Insight into the Structure–Activity Relationship in Electrochromism of W0_3_ with Rational Internal Cavities for Broadband Tunable Smart Windows." *Chemical Engineering Journal* 470 (2023): 144130, https://doi.org/10.1016/j.cej.2023.144130

[3]	Xing, C.; Yang, L.; He, R.; Spadaro, M. C.; Zhang, Y.; Arbiol, J.*; et al.* "Brookite TiO_2_ Nanorods as Promising Electrochromic and Energy Storage Materials for Smart Windows." *Small* 19 (2023): 2303639, https://doi.org/10.1002/smll.202303639

[4]	Kim, J.; Haeng Lee, K.; Seo, D.-B.; Jang, H.; Kang, S.; Yim, S.*; et al.* "Two-Dimensional VO_2_ Nanosheet Converted from Mxene for Flexible Thermochromic Smart Windows." *Chemical Engineering Journal* 477 (2023): 147014, https://doi.org/10.1016/j.cej.2023.147014

[5]	He, H.; Liu, S.; Du, Y.; Zhang, R.; Ho, T. C.; Tso, C. Y. "Enhancing Stability: Two-Dimensional Thermochromic Perovskite for Smart Windows in Building Applications." *Advanced Functional Materials* 36 (2026): 2417582, https://doi.org/10.1002/adfm.202417582

[6]	Wang, S.; Zhou, Y.; Jiang, T.; Yang, R.; Tan, G.; Long, Y. "Thermochromic Smart Windows with Highly Regulated Radiative Cooling and Solar Transmission." *Nano Energy* 89 (2021): 106440, https://doi.org/10.1016/j.nanoen.2021.106440

[7]	Mohammad, N. M.; Zhang, Y.; Xu, W.; Aranke, S. S.; Carne, D.; Deng, P.*; et al.* "Highly Tunable Cellulosic Hydrogels with Dynamic Solar Modulation for Energy-Efficient Windows." *Small* 20 (2024): 2303706, https://doi.org/10.1002/smll.202303706

[8]	Zhang, L.; Du, Y.; Xia, F.; Gao, Y. "Two Birds with One Stone: A Novel Thermochromic Cellulose Hydrogel as Electrolyte for Fabricating Electric-/Thermal-Dual-Responsive Smart Windows." *Chemical Engineering Journal* 455 (2023): 140849, https://doi.org/10.1016/j.cej.2022.140849

[9]	Lee, S. J.; Lee, S. H.; Kang, H. W.; Nahm, S.; Kim, B. H.; Kim, H.*; et al.* "Flexible Electrochromic and Thermochromic Hybrid Smart Window Based on a Highly Durable ITO/Graphene Transparent Electrode." *Chemical Engineering Journal* 416 (2021): 129028, https://doi.org/10.1016/j.cej.2021.129028

[10]	Charbonnel, T.; Prontera, C. T.; Pugliese, M.; Di Masi, S.; Giuri, A.; Cannavale, A.*; et al.* "Multifunctional Thermo-Electrochromic Devices Based on 2D Perovskite Gel Electrolyte." *Nano Energy* 142 (2025): 111271, https://doi.org/10.1016/j.nanoen.2025.111271
